# *Boesenbergia rotunda*–Derived Phytochemicals as Potent Inhibitors of SARS‐CoV‐2 Papain–Like Protease (PLpro): Insights From Molecular Docking and Dynamic Simulation

**DOI:** 10.1155/sci5/1695824

**Published:** 2026-04-29

**Authors:** Dhrubo Ahmed Khan, Mohammad Ashik Sheikh, Raihan Rahman Imon, Md. Imtiaz, Shamin Ahmed, Harasit Gharami, Ryan V. Labana, Tajudeen O. Jimoh, Anand Gaurav, Maria L. Pereira, Muhammad Nawaz, Julieta Z. Dungca, Md. Nazmul Hasan, Manik Ghosh, Veeranoot Nissapatorn

**Affiliations:** ^1^ Department of Genetic Engineering and Biotechnology, Faculty of Biological Science and Technology, Jashore University of Science and Technology, Jashore, 7408, Bangladesh, just.edu.bd; ^2^ Department of Pharmacy, Faculty of Biological Science and Technology, Jashore University of Science and Technology, Jashore, 7408, Bangladesh, just.edu.bd; ^3^ Center for Health Sciences, Research Institute for Science and Technology, Polytechnic University of the Philippines, Sta. Mesa, Manila, 1008, Philippines, pup.edu.ph; ^4^ Department of Biology, College of Science, Polytechnic University of the Philippines, Sta. Mesa, Manila, 1008, Philippines, pup.edu.ph; ^5^ Department of Microbiology, Icahn School of Medicine at Mount Sinai, New York, New York, USA, mountsinai.org; ^6^ Department of Pharmaceutical Sciences, School of Health Sciences and Technology, UPES, Dehradun, 248007, Uttarakhand, India, upes.ac.in; ^7^ Department of Medical Sciences & CICECO-Aveiro Institute of Materials, University of Aveiro, Aveiro, Portugal, ua.pt; ^8^ Department of Nano-Medicine Research, Institute for Research and Medical Consultations (IRMC), Imam Abdulrahman Bin Faisal University, Dammam, Saudi Arabia, iau.edu.sa; ^9^ School of Science and Technology, Centro Escolar University, 9 Mendiola St. San Miguel, Manila, 1005, Philippines; ^10^ Department of Pharmaceutical Sciences & Technology, Birla Institute of Technology, Mesra, Ranchi, 835 215, Jharkhand, India, bitmesra.ac.in; ^11^ School of Allied Health Sciences and World Union for Herbal Drug Discovery (WUHeDD), Walailak University, Nakhon Si Thammarat, Thailand, wu.ac.th

## Abstract

The papain‐like protease (PLpro) of SARS‐CoV‐2 plays fundamental roles in its replication, and its mechanistic inhibition can impede the virus’s replication and infection. Most Plpro inhibitors identified thus far are chemically synthesized and subject to numerous restrictions regarding stability and adverse side effects. Nevertheless, the inhibitors of those compounds can be replaced with natural, selective PLpro inhibitors that are highly stable and have minimal adverse effects. Since ancient times, extracts of *Boesenbergia rotunda* (L.) Mansf. have been recognized for their antiviral and other properties. Consequently, the objective of the investigation was to investigate the inhibitory activity of *B. rotunda* extract compounds against the virus, with the intention of inhibiting PLpro’s signaling function in its replicative pathway, as a result, preventing viral infections. Molecular docking was initially suggested to evaluate the level of binding affinity among 57 natural compounds identified from *B. rotunda* to the desired protein. The results of this computational analysis have additionally been compared against molnupiravir, which has been addressed experimentally for its interacting efficiency towards the PLpro receptor protein of SARS‐CoV‐2 lately. This comparison indicates that the proposed dietary compounds have a significantly noticeable interaction efficiency regarding binding efficiency and other energetic contributions. Furthermore, the structure of PLpro was significantly influenced by compounds in MD‐simulation experiments that were validated through some standard analyses, such as RMSF (root mean square fluctuation), RMSD (root mean square deviation), solvent accessible surface area, radius of gyration, MolSA, and PSA. The most promising three phytochemicals that could be established as an antiviral curative option against SARS‐CoV‐2 infection have been identified through computational approaches: rubranine, boesenbergin B, and panduratin A. The results of our computational investigation indicate that our proposed medications require clinical experimentation; consequently, they may be a superior treatment against SARS‐CoV‐2 viral infection.

## 1. Introduction

The very recent deadliest virus, Severe Acute Respiratory Syndrome Coronavirus 2 (SARS‐CoV‐2), has been classified in the Coronaviridae family by the International Committee on Taxonomy of Viruses organization’s responsible scientific panel, CSG (Coronaviridae Study Group) [1]. The SARS‐CoV‐2 virus resembles a corona (Latin for “crown”) due to its central RNA genome surrounded by an envelope studded with spike‐like glycoproteins, which inspired the name “coronavirus” [2]. Coronaviruses are zoonotic viruses that are transmitted from an animal host to humans via an intermediary second animal host [3]. As reviewed by Lima [4], the major characteristics of SARS‐CoV‐2 infection are a high body temperature, coughing, muscle soreness, sore throat, headache, breathing difficulties, mental disorientation, chest pain, rhinorrhea, vomiting/nausea, and diarrhea, which can lead to serious pneumonia [5].

The recent COVID‐19 pandemic, named after the coronavirus disease of 2019, has been caused by SARS‐CoV‐2, which first appeared in China’s Wuhan city around the end of 2019 [6]. Within a short period, the virus, as of October 28, 2022, infected 634,784,361 people worldwide, leading to 6,589,972 deaths and a world average of 81,437 infected cases per million population [7]. Among the most affected Southeast Asian countries, Indonesia has 6,484,764 infected cases, 158,544 total deaths, and 23,232 cases per million population. In Malaysia, total infected cases run to 4,890,437, and total deaths and cases per million population are 36,462 and 147,386. In Thailand, the respective figures are 4,689,897; 32,922; and 66,924. Treatment and prevention of COVID‐19 cases are putting a severe economic burden on both governments and families, the burden being accentuated by lockdowns and the closing of offices, businesses, and educational institutions.

Several vaccines against SARS‐CoV‐2, such as Moderna, Oxford–AstraZeneca, Pfizer–BioNTech, SinoVac, Johnson & Johnson, and Sputnik V, developed in many countries, have been approved by the WHO (World Health Organization) on an emergency basis. In the meantime, SARS‐CoV‐2 has evolved into numerous variants, which may compromise vaccine efficacy [8].

Antiviral drugs could be an effective treatment method against SARS‐CoV‐2 viral infection and its numerous variants. Several FDA (Food and Drug Administration)–U.S.–approved drug candidates have been repurposed and used to treat COVID‐19 [9]. Among these drugs, lopinavir/ritonavir are widely used as human immunodeficiency virus (HIV) drugs; oseltamivir is another one that is used to treat flu; to treat Ebola patients, remdesivir has been utilized lately; and favipiravir, darunavir, and umifenovir have been recently exhibited to possess anti‐SARS‐CoV‐2 properties. Molnupiravir is one of the latest drugs approved for treating COVID‐19 infections. An antiviral drug (previously known as EIDD‐2801), a prodrug of β‐D‐N4‐hydroxycytidine (EIDD‐1931), has been efficient in inhibiting the RNA‐dependent RNA polymerase in SARS‐CoV‐2 replication. The drug is used against early and mild COVID‐19 patients [10]. However, these therapeutics’ efficiency level and long‐term complications are still being debated [11]. Other repurposed drugs include ribavirin, interferons, and hydroxychloroquine; however, inadequate effectiveness has been shown in the initial open‐label randomized controlled trials [12].

The present scenario calls for a “return to the basics” to find novel plant‐based drugs and lead compounds for treating COVID‐19 as a priority and for viral diseases caused by SARS and MERS in case they re‐emerge. Plants have always been a major source of discovery for new drugs. [13]. Secondary metabolites, often known as phytochemicals, are produced by plants and have a variety of pharmacological characteristics that can be used by scientists to cure a wide range of illnesses, either with or without additional changes to the phytochemicals’ carbon skeletons. Phytochemicals and plant‐derived extracts can be useful for identifying any promising active components against SARS‐CoV‐2 [14].

*Boesenbergia rotunda* (L.) Mansf. (Zingiberaceae) is known in English as fingerroot. Its synonym is *Kaempferia pandurata* Roxb. The plant is considered a medicinal and culinary herb in China and Southeast Asian countries. The phytochemicals and antiviral activities of *B. rotunda* (another synonym of *Boesenbergia pandurata* (Roxb.)) have been quite elaborately reviewed by Eng‐Chong and others [15]. The rhizomes of the plant are a rich source of various flavonoid compounds. *B. rotunda* is rich in chalcones and flavonoids, a group of compounds. Both of these groups of compounds are effective in silico/computational or in vitro or in vivo investigations against several virus species, including SARS‐CoV‐2 [16–18].

The current issue with herbs and their phytochemicals is that, despite the abundance of computational and in vitro studies showing potential phytochemicals that inhibit SARS‐CoV‐2 protein, including papain‐like protease (PLpro) [19–21], no herbal‐derived drug has been clinically established through appropriate trials to inhibit viral replication or its activity. This highlights the necessity for additional in vitro, in silico, and in vivo research to find new treatments against SARS‐CoV‐2 [22]. This research aims to describe drug target discovery studies that identify one highly promising candidate plant in Southeast Asia and its phytochemicals that may be useful in treating COVID‐19, mostly in silico analyses of a particular plant metabolite [23].

## 2. Methods and Materials

### 2.1. Construction of a Phytochemical Library

The accessible compounds from the selected medicinal plant were identified using the Indian Medicinal Plants, Phytochemistry, and Therapeutics (IMPPAT) compound library [24], and the corresponding phytochemical information was retrieved from the PubChem database [25]. For in silico analysis, a phytochemical library consisting of 57 bioactive compounds was compiled. To evaluate the pharmacokinetic and toxicity profiles (ADMET: absorption, distribution, metabolism, excretion, and toxicity), two widely used online tools were employed. The drug‐like properties of selected compounds were examined based on the “Lipinski Rule of Five” to allow the identification of potential antiviral lead molecules [26]. The pharmacokinetic characteristics of these compounds were evaluated using the Swiss ADME and pkCSM servers [27, 28], and parameters such as lipophilicity, plasma protein binding, water solubility, and drug‐likeness were assessed.

### 2.2. Blind Docking Methodology

#### 2.2.1. Receptor Preparation

After removing an inhibitor as our target receptor, we used the PLpro crystal structure of SARS‐CoV‐2. The protein’s X‐ray crystallographic structure from the database Protein Data Bank (PDB), holding the PDB ID‐6W9C, was obtained afterward. To prepare this crystallographic protein structure for docking, we eliminated the molecules of water and added polar hydrogen atoms due to their usual absence in crystallographic structures. Polar hydrogen atoms were added along with the removal of water molecules, with the assistance of UCSF Chimera 1.14 [29]. The prepared protein molecule was then saved in PDB format.

#### 2.2.2. Docking Parameters

Autodock Vina 1.2 was used for molecular docking, and a grid box was generated with appropriate dimensions and coordinates to cover the whole protein structure [30]. Grid box parameters: The grid box’s dimensions were *X*: 64.59, *Y*: 45.69, and *Z*: 65.88 (unit: Angstrom/Å), along with the location of the center point at *X*: −30.04, *Y*: 34.02, and *Z*: 24.60.

#### 2.2.3. Ligand Selection and Preparation

The SDF format of selected ligand structures was obtained from the PubChem database [31]. The ligand geometries and their charges had been optimized using UCSF Chimera 1.14 and saved in PDBQT format. The well‐known antiviral compound molnupiravir (PubChem CID‐145996610) was selected to serve as the control for the docking and molecular dynamics (MD) studies [32].

#### 2.2.4. Molecular Docking and Visualization

A blind docking technique was implemented to experiment with plant metabolites against the COVID‐19 PLpro to identify potentially suitable inhibitors. Thus, as described previously, the grid box in AutoDock Vina 1.2 was created to encompass the entire protein molecule. The regions where these ligands bind with the target protein can be identified using blind docking; new binding pockets can also be potentially revealed during the process. Exhaustiveness was set at “51” so that more accurate results in binding energy and interactions can be obtained. AutoDock Vina produces nine docked poses for every ligand, where the best pose was pose 1, along with its greatest binding affinity [30]. LigPlot + Version 2.2 analyzed the polar and hydrophobic noncovalent interactions among protein–ligand complexes [33]. The ligand–protein complex files were generated using PyMOL [34]. Furthermore, the compactness of the complexes’ structural bonding, along with their polar and nonpolar interaction bonds, was validated using BIOVIA Discovery Studio Visualizer 64‐bit.

### 2.3. Prime‐Based MM–GBSA Free Energy Estimation

The representative protein–ligand complex, which was previously docked using AutoDock Vina, served as the initial structure for our study. Ligand coordinates were extracted using Maestro (Schrödinger Suite 2024‐4), resulting in the selection of four distinct docked conformations. To define a consistent docking region, we calculated the spatial centroid and coordinate range of these ligand poses. A receptor grid was then created based on the midpoint of the *x*, *y*, and *z* coordinates, with an additional 10 Å buffer applied in each dimension. This approach ensured complete coverage of all conformers while allowing for structural flexibility during the docking process. After generating the grid, extra precision (XP) docking was performed using the Glide module for all test compounds, including known controls. The resulting docked complexes underwent postdocking binding energy analysis utilizing the Prime MM–GBSA approach (Schrödinger Suite 2024‐4). The MM–GBSA calculations utilized the OPLS4 force field along with the VSGB 2.1 implicit solvent model [35]. The binding free energy (Δ*G*_bind) for each ligand–receptor complex was calculated using the following equation:

(1) ΔG bind=Ecomplexmin−Eligandmin+Ereceptormin.

In this equation, each energy term reflects the minimized energy of the respective species. This includes considerations for van der Waals interactions, such as π–π stacking and self‐contact, as well as electrostatic contributions like Coulombic forces, hydrogen bonding, and solvation effects, which are modeled using the Generalized Born approximation. The strain energies of both the ligand and receptor were assessed to evaluate the conformational penalties that occur upon binding. Overall, the Δ*G* bind values obtained from MM–GBSA were utilized to rank the ligand candidates, where more negative values indicate higher binding affinity and stronger molecular interactions [36–38].

### 2.4. MD Simulation

A 100 ns MD simulation was applied for the determination of protein–ligand complexes’ stability and binding dynamics [39–41]. Schrödinger LLC’s Desmond v3.6 simulation package [42] in a Linux environment was used to perform MD simulations [43, 44]. The preparation of simulation systems was done with the assistance of the System Builder tool, “Desmond.” An appropriate number of Na^+^ or Cl^-^ counterions were used to neutralize every system, and water molecules were explicitly defined (TIP3P). For every system, an orthorhombic simulation box was applied with periodic boundary conditions (PBCs) and a 10 Å space between the solute and the edge of the box. Minimization and equilibration of the simulation systems were performed sequentially before the real simulations and before the production simulation. The Nosé–Hoover chain thermostat [45] was used to set the temperature at 300 K along with a relaxation period of 1.0 ps, while with the help of the Martyna–Tobias–Klein barostat [37], isotropic coupling, and a relaxation period of 2.0 ps, the pressure was set at 1.0 bar.

#### 2.4.1. Simulation Trajectory Analysis

Application v9.5 of the Maestro program was utilized to study simulation trajectories. The implemented Simulation Interaction Diagram tool on Desmond was used for the assessment of dynamic interactions within proteins and ligands. The stability of ligand–protein complexes was evaluated by analyzing the RMSD (root mean square deviation) of the ligand and protein atomic positions over time. Solvent accessible surface area (SASA), radius of gyration (Rg), molecular surface area (MolSA), intramolecular hydrogen bonds, and polar surface area (PSA) of the structure of a protein–ligand complex were also studied [39].

### 2.5. Post‐MD Thermal MM–GBSA Calculation

During simulation, we used the thermal_mmgbsa.py Python program to determine the binding free energies of the complexes. Twenty distinct frame snapshots of the Desmond MD trajectory were taken, and MM–GBSA analysis was performed on each frame. The ligand and receptor were then separated for further study [46].

## 3. Results

### 3.1. Pharmacokinetic and Toxicity Analysis of the Selected Ligands

**FIGURE 1:**
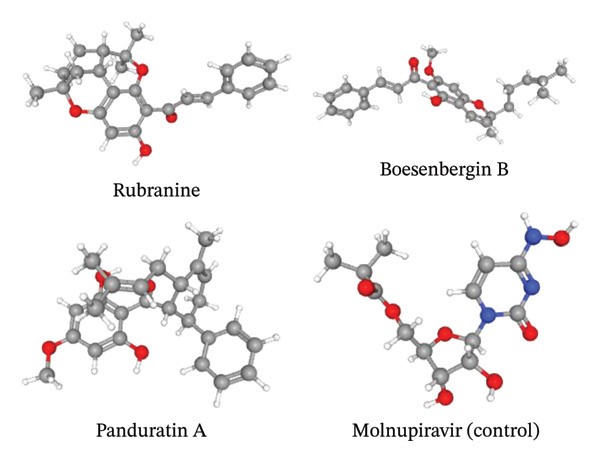
The chemical structures of three phytochemicals (rubranine, boesenbergin B, and panduratin A), as well as the control compound molnupiravir, are illustrated.

The phytochemicals chosen for this study were assessed for toxicity and pharmacokinetic properties using the SwissADME and pkCSM platforms, taking into account parameters such as molecular weight, LogP, hydrogen bond donors and acceptors, rotatable bonds, absorption, metabolism, excretion, and toxicity. The compounds were evaluated according to Lipinski’s rule of five and standard drug‐likeness filters. Ultimately, rubranine, boesenbergin B, and panduratin A were selected for further docking studies due to their high intestinal absorption, nontoxic profiles, and favorable drug‐likeness properties (Table 1, Figure 1).

**TABLE 1:** Pharmacophore and pharmacokinetic profile for the selected ligand molecules.

Ligands name	MW	NHA	NHD	LogP	NRB	IA	TC	LD50	BBB	HT	AT	MTD	NLV	DL
Molnupiravir	329.309	10	4	−1.1372	5	53.464	0.203	2.158	NO	YES	NO	0.28	NO	YES
Rubranine	390.479	4	1	5.4941	3	94.068	0.152	2.539	YES	NO	NO	−0.156	NO	YES
Boesenbergin B	404.506	4	1	6.2076	7	93.896	0.171	2.303	NO	NO	NO	0.982	NO	YES
Panduratin A	406.522	4	2	6.0116	6	91.308	0.266	2.288	NO	NO	NO	0.941	NO	YES

*Note:* NHA, no. of hydrogen bond acceptors; NHD, no. of hydrogen bond donors; LogP, predicted octanol/water partition coefficient; LD50, oral rat acute toxicity; HT, hepatotoxicity; MTD, maximum tolerated dose for human (log mg/kg/day).

Abbreviations: AT, AMES toxicity; BBB, blood–brain barrier; DL, drug likeness; IA, intestinal absorption (% absorbed); MW, molecular weight (g/mol); NLV, number of Lipinski’s violations; NRB, no. of rotatable bonds; TC, total clearance (log mL/min/kg).

### 3.2. Interpretation of Molecular Docking

Molecular docking is a simulation technique that has been used to predict how a macromolecule, such as a receptor protein, interacts with a small, drug‐like molecule [43]. A molecular docking approach was employed utilizing Auto Dock Vina software to determine the binding affinity between active site residues of the PLpro protein and selected phytochemical ligands (Figure 2). Molnupiravir, a control for comparison with the investigated ligands, exhibited a binding free energy of −6.2 kcal/mol towards PLpro. Table 1 presents a summary of the binding free energies of the selected compounds that were studied. The greatest binding affinity (minimum binding free energy) was demonstrated by rubranine (−8.2 kcal/mol), while boesenbergin B and panduratin A followed closely with −7.4 and −7.3 kcal/mol. All three showed binding affinity higher than the control.

FIGURE 2(a) The interactions involving postdocking analysis of SARS‐CoV‐2 PLpro (PDB ID: 6W9C) in complex with the selected ligands rubranine and boesenbergin B are represented. (b) The interactions involving postdocking analysis of SARS‐CoV‐2 PLpro (PDB ID: 6W9C) in complex with the selected ligands panduratin A and control molnupiravir are represented.

**Figure:**
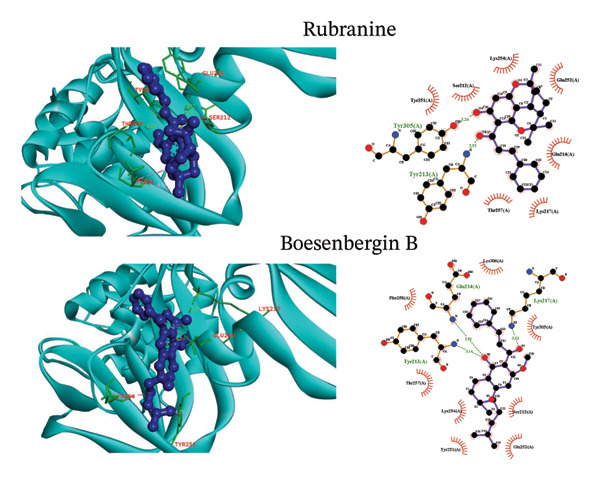
(a)

**Figure:**
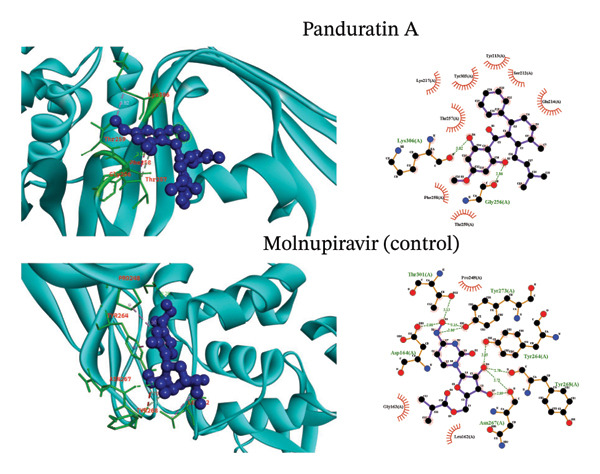
(b)

### 3.3. Visualization of Postdocking Protein–Ligand Interactions

LigPlot + Version 2.2 and BIOVIA Discovery Studio Visualizer were utilized to analyze interactions among the targeted viral protein and selected phytochemical ligands. Table 2 and Figure 2 present the results of the LigPlot + analysis of molecular interactions, mainly hydrophobic and hydrogen or noncovalent bonds.

**TABLE 2:** The molecular interactions between selected phytochemicals and the targeted SARS‐CoV‐2 PLpro are reported in the docking score.

Compounds	Docking score (kcal/mol)	Amino acid participation in bonding interaction	
		Interaction of the hydrogen bond	Interaction of the hydrophobic bond
Rubranine (CID‐42607681)	−8.2	Tyr305 (3.24 Å), Tyr 213 (2.91 Å)	Tyr251, Ser212, Lys254, Glu252, Glu214, Lys217, Thr 257
Boesenbergin (CID‐23643133)	−7.4	Tyr213 (3.14 Å), Glu214 (2.91 Å), Lys217 (3.32 Å)	Phe258, Lys306, Tyr305, Ser212, Glu252, Tyr251, Lys254, Thr257
Panduratin (CID‐6483648)	−7.3	Lys306 (3.03 Å), Gly256 (2.86 Å)	Thr257, Lys217, Tyr305, Tyr213, Ser212, Glu214, Thr259, Phe258
Molnupiravir (CID‐145996610) control	−6.2	Thr301 (3.13 Å), Asp164 (2.88 Å), Tyr273 (3.15, 2.86 Å), Tyr264 (2.85 Å), Tyr268 (2.70 Å), Asn267 (2.89, 2.75 Å)	Pro248, leu162, Gly163

Molnupiravir (CID‐145996610), the control for this study, showed eight hydrogen bonds with Thr301 (3.13 Å), Asp164 (2.88 Å), Tyr273 (3.15, 2.86 Å), Tyr264 (2.85 Å), Tyr268 (2.70 Å), and Asn267 (2.89, 2.75 Å) and three hydrophobic bonds (with Pro248, leu162, and Gly163) with the PLpro. The PLpro protease–rubranine complex involved two hydrogen bonds (Tyr305 (3.24 Å), Tyr 213 (2.91 Å)) and seven hydrophobic bonds (Tyr251, Ser212, Lys254, Glu252, Glu214, Lys217, and Thr 257) (Figure 2), whereas boesenbergin formed three hydrogen bonds (Tyr213 (3.14 Å), Glu214 (2.91 Å), Lys217 (3.32 Å)) and eight hydrophobic bonds (Phe258, Lys306, Tyr305, Ser212, Glu252, Tyr251, Lys254, and Thr257) PLpro. Panduratin showed similar interactions, that is, two hydrogen bonds (Lys306 (3.03 Å), Gly256 (2.86 Å)) and eight hydrophobic bonds (Thr257, Lys217, Tyr305, Tyr213, Ser212, Glu214, Thr259, and Phe258).

### 3.4. Binding Affinity Assessment Using the MM–GBSA Method

**FIGURE 3:**
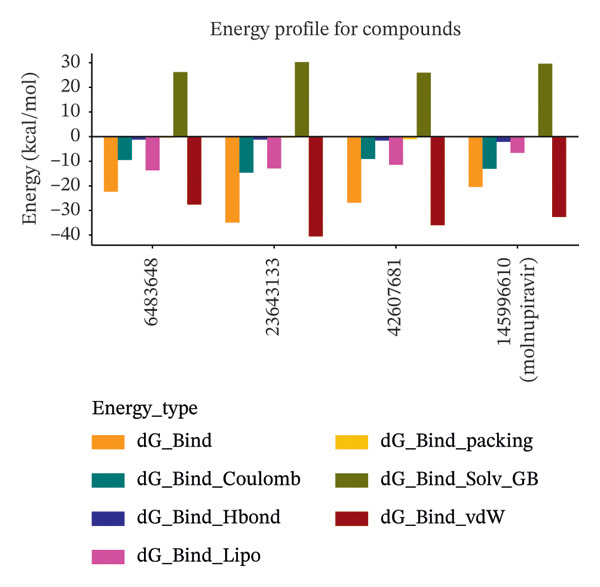
Bar graph illustrating the MM–GBSA energy component profiles for each ligand compared to molnupiravir. Energy terms are color‐coded using the following scheme: ΔG_Bind in orange, ΔG_Bind_Coulomb in teal, ΔG_Bind_Hbond in navy, ΔG_Bind_Lipo in magenta, ΔG_Bind_Packing in gold, ΔG_Bind_Solv_GB in olive, and ΔG_Bind_vdW in maroon. All energy values are expressed in kcal/mol.

The binding free energy (ΔG bind) and its energy components were computed for four protein–ligand complexes using the Prime MM–GBSA method. The results are summarized in Table 3 and Figure 3. Compared to molnupiravir (CID 145996610), which showed a ΔG Bind of −20.50 kcal/mol, all three test compounds exhibited more favorable binding energies. The most favorable binding energy was observed for CID 23643133 with a ΔG Bind of −35.08 kcal/mol, which is 14.58 kcal/mol lower than that of molnupiravir. This was followed by CID 42607681 (−27.04 kcal/mol) and CID 6483648 (−22.55 kcal/mol), showing ΔG Bind improvements of 6.54 and 2.05 kcal/mol, respectively, relative to molnupiravir.

**TABLE 3:** MM–GBSA binding free energies and energy components (kcal/mol) for candidate ligands and molnupiravir (control).

CID	ΔG Bind (kcal/mol)	ΔG Bind Coulomb (kcal/mol)	ΔG Bind Hbond (kcal/mol)	ΔG Bind Lipo (kcal/mol)	ΔG Bind Packing (kcal/mol)	ΔG Bind Solv GB (kcal/mol)	ΔG Bind vdW (kcal/mol)
6483648	−22.55	−9.55	−1.16	−13.74	−0.55	26.06	−27.76
23643133	−35.08	−14.46	−1.23	−13.12	−0.69	30.10	−40.64
42607681	−27.04	−9.18	−1.50	−11.68	−1.13	26.07	−36.13
145996610 (molnupiravir)	−20.50	−13.17	−2.24	−6.71	−0.17	29.51	−32.85

The van der Waals energy (ΔG vdW) contributions were −40.64, −36.13, −27.76, and −32.85 kcal/mol for CID 23643133, 42607681, 6483648, and molnupiravir, respectively. Coulombic interactions (ΔG Coulomb) ranged from −9.18 to −14.46 kcal/mol across all ligands. Hydrogen bonding energies (ΔG Hbond) were between −1.16 and −2.24 kcal/mol, with the lowest value observed for molnupiravir. Lipophilic interactions (ΔG Lipo) ranged from −6.71 kcal/mol (molnupiravir) to −13.74 kcal/mol (CID 145996610). Packing energies (ΔG Packing) were modest, ranging from −0.17 to −1.13 kcal/mol. Solvation penalties (ΔG Solv GB) were positive for all ligands, with values of 26.06, 30.10, 26.07, and 29.51 kcal/mol, respectively.

### 3.5. MD Simulation

In computational drug discovery, MD simulation is frequently used to investigate the dynamics and stability of intermolecular interactions among target proteins and selected ligands in a complex. A 100 ns MD simulation for all atoms was used to investigate the dynamic behavior and the interaction stability of the PLpro receptor protein with the attached ligands being studied, including the control molnupiravir. The RMSD, root‐mean‐square fluctuation (RMSF), Rg, MolSA, SASA, PSA, and intramolecular hydrogen bonding had been used to examine the MD simulation results (Intra HB).

#### 3.5.1. RMSD

**FIGURE 4:**
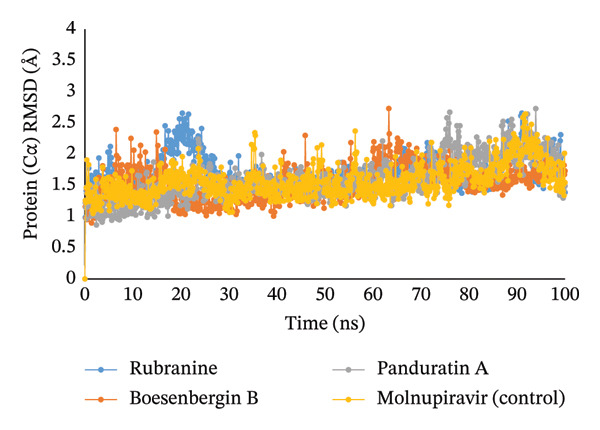
The RMSD of 100 ns MDS for the PLpro (PDB ID: 6W9C) protein of SARS‐CoV‐2 in complex with the four ligands rubranine, boesenbergin B, panduratin A, and control molnupiravir, indicated by blue, orange, gray, and yellow colors, respectively.

The RMSD value of the atoms in the PLpro–ligand complexes was observed during the 100 ns MD simulation. It is acceptable for the average or mean value to vary between frames within a range of 1–3 Å or 0.1–0.3 nm; greater values than this intended range suggest a substantial structural change in the protein. All the ligands being studied, that is, rubranine (PubChem CID‐42607681), boesenbergin B (PubChem CID‐23643133), and panduratin A (PubChem CID‐6483648), showed considerable stability with a standard range of RMSD value between 1 and 3 Å in MD simulation. Rubranine showed initial fluctuation but stabilized over time and exhibited an acceptable RMSD value (Figure 4).

#### 3.5.2. RMSF

**FIGURE 5:**
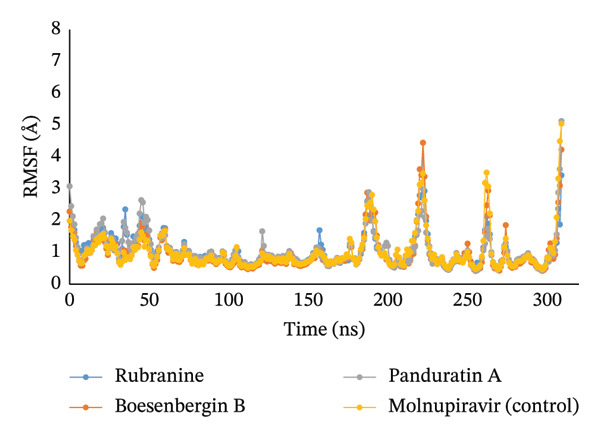
The RMSF values for the protein C atoms of the PLpro (PDB ID: 6W9C) protein of SARS‐CoV‐2 in complex with the selected ligands rubranine, boesenbergin B, panduratin A, and control molnupiravir are indicated by blue, orange, gray, and yellow colors, respectively.

For the characterization and identification of local changes in the targeted protein chain caused by chemicals interacting with specific residues, RMSF could be a good option. The RMSF value is essential for characterizing a protein, as it provides information on both local alterations and the behavior of the protein chain. The RMSF for the selected natural compounds rubranine, boesenbergin B, panduratin A, and control molnupiravir was determined using the residue C index from the complex with SARS‐CoV‐2’s protein PLpro (PDB ID: 6W9C), as shown in Figure 5. The RMSF values of the compounds CID‐42607681, CID‐119245, CID‐156783, and control CID‐145996610 in complex with the PLpro (PDB ID: 6W9C) protease were determined to examine how the binding of specific ligand compounds to a particular residue alters the protein’s structural flexibility. When comparing the compounds rubranine, boesenbergin B, and panduratin A to the control molnupiravir, the RMSF graph exhibited little fluctuations between 185–193, 217–224, 260–265, and 306–309 residues within the 5.045 maximum range, which is completely acceptable. Because the C‐ and N‐terminal domains are present, most of the fluctuation occurs at the beginning and end of the protein. As a result, for all three compounds studied, the displacement of a specific atom has a lower fluctuation possibility in a real‐life context.

#### 3.5.3. The Rg Analysis

**FIGURE 6:**
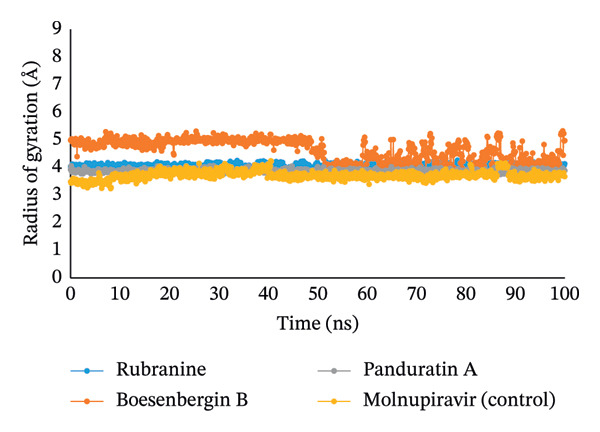
The radius of gyration (Rg) value of 100 ns MDS analyses for the PLpro (PDB ID: 6W9C) protein of SARS‐CoV‐2 in complex with the selected ligands rubranine, boesenbergin B, panduratin A, and control molnupiravir, indicated by blue, orange, gray, and yellow colors, respectively.

To examine the center of gravity, we determine the Rg value for the protein backbone, which indicates the compactness of the protein and provides an overall framework for the distribution of molecules within it. The Rg is one of the principal indicators for predicting the structural activity of macromolecules because it reflects changes in complex compactness. As shown in Figure 6, the stability of the compounds rubranine, boesenbergin B, panduratin A, and the control molnupiravir in interaction with the PLpro protease was examined concerning Rg throughout a 100‐ns simulation period. Boesenbergin B displayed the highest rate of variations, while molnupiravir exhibited the lowest rate. Meanwhile, the other compounds, rubranine (CID‐42607681) and panduratin A (CID‐6483648), showed minimal and similar results during the 100 ns simulation process. The mean Rg values of the phytochemical ligands rubranine, boesenbergin B, panduratin A, and the control molnupiravir were 4.02, 4.73, 3.76, and 3.65, respectively. This investigation demonstrates that when the selected ligand phytochemicals are bound to the targeted protein, the binding site of this protein does not change significantly.

#### 3.5.4. Analysis of SASA, MolSA, and PSA

**FIGURE 7:**
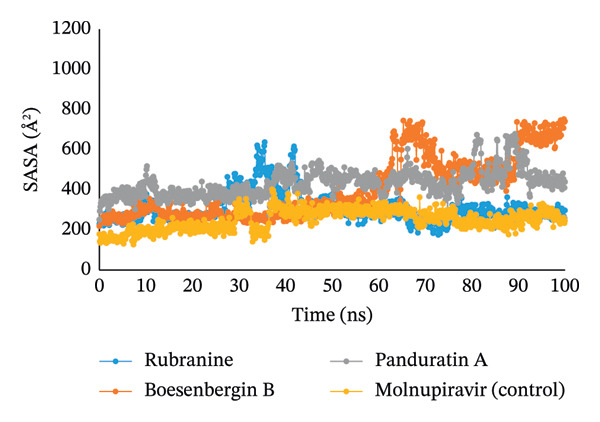
The radius of SASA values of 100 ns MDS evaluations for PLpro (PDB ID: 6W9C) protein of SARS‐CoV‐2 in complex with the selected ligands rubranine, boesenbergin B, panduratin A, and control molnupiravir indicated by blue, orange, gray, and yellow colors, respectively.

The simulated trajectories’ SASA (solvent‐accessible surface area) was examined to explain the protein’s conformational alteration. Furthermore, its analysis validates the results from RMSD, RMSF, and Rg analyses. According to the SASA analysis, the complex’s structural conformation has changed, with a few residues in the protein’s core now exposed to solvent. A protein’s surface may have active sites and/or ligand‐binding sites, which can be used to better understand the protein’s solvent‐like properties (hydrophilic or hydrophobic) and interactions between the target protein and ligands. With little fluctuations, compound panduratin A has the highest SASA profile. The compounds, such as rubranine, had a consistent SASA profile with minor deviations, similar to the molnupiravir control drug in Figure 7. The SASA value for another compound, boesenbergin B, showed slight fluctuation in the 100 ns simulation process at the terminal period, showing that amino acid residues in the complex systems had been exposed to significant concentrations of the selected ligand compounds.

**FIGURE 8:**
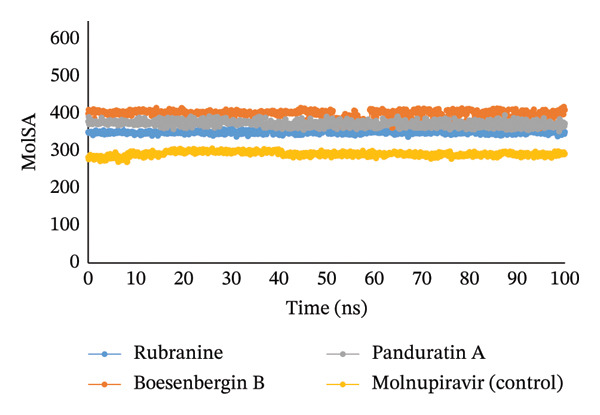
The MolSA values of 100 ns MDS evaluations for the PLpro (PDB ID: 6W9C) protein of SARS‐CoV‐2 in complex with the selected ligands rubranine, boesenbergin B, panduratin A, and the control molnupiravir, indicated by blue, orange, gray, and yellow colors, respectively.

The MolSA and the van der Waals surface area are equivalent when the probe radius is set to 1.4. All ligand compounds—rubranine, boesenbergin B, panduratin A, and control molnupiravir with PLpro protease—had demonstrated the standard van der Waals surface area in our computational analysis, as illustrated in Figure 8.

**FIGURE 9:**
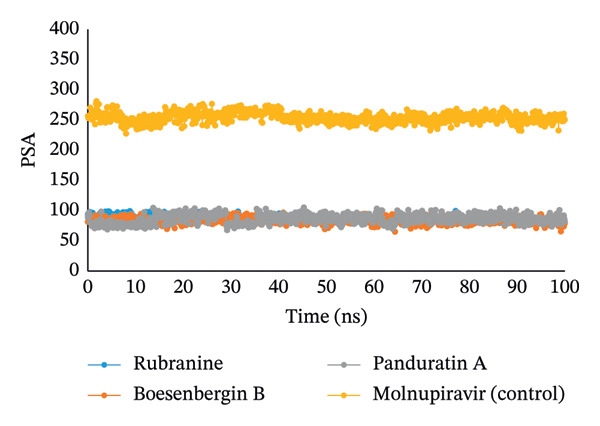
The radius of PSA values of 100 ns MDS evaluations for PLpro (PDB ID: 6W9C) protein of SARS‐CoV‐2 is complex with the selected ligands rubranine, boesenbergin B, panduratin A, and control molnupiravir represented by blue, orange, gray, and yellow colors, respectively.

Furthermore, only the oxygen and nitrogen atoms determine a structure’s PSA. All ligands, rubranine, boesenbergin B, panduratin A, and the control molnupiravir with the protein PLpro had a high PSA value, as shown in Figure 9.

#### 3.5.5. Analysis of Intramolecular Bonds

**FIGURE 10:**
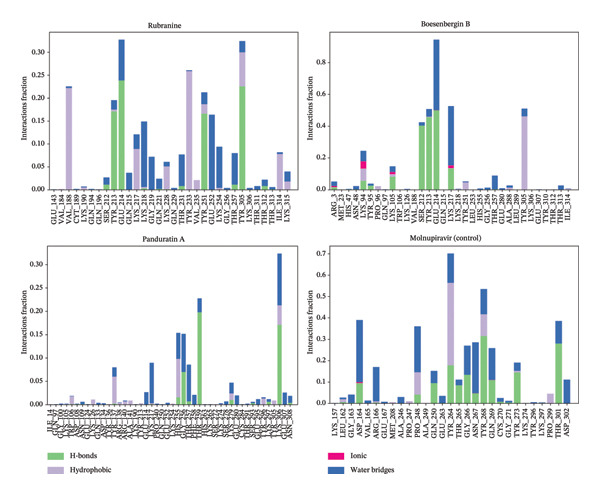
The interactions involving intramolecular bonds of 100 ns MDS evaluations for PLpro (PDB ID: 6W9C) protease of SARS‐CoV‐2 in the complex with the selected ligands rubranine, boesenbergin B, panduratin A, and control molnupiravir are represented.

The hydrogen bonds, hydrophobic interactions, and water bridge bonds in protein–ligand interactions influence drug selectivity, metabolism, and absorption. The interaction between the PLpro (PDB ID: 6W9C) target protein and the selected phytochemical compounds—rubranine, boesenbergin B, panduratin A, and the control molnupiravir—has been analyzed using various elements, including hydrogen bonds, noncovalent (hydrophobic) bonds, ionic bonds, and water bridge bonds, as depicted in Figure 10. In the compounds rubranine, boesenbergin B, panduratin A, and the control molnupiravir with the protein, multiple bonds created the maximum interaction fraction value. The most stable are the compounds with the greatest number of hydrogen bonds and water bridges, as well as the compounds rubranine and control molnupiravir. Significant hydrogen bonds were formed by all the bioactive phytochemicals simulated with protein residues. Each compound was found to form numerous contacts by ionic, hydrophobic, hydrogen, and water bridge bonds, which were sustained until the conclusion of the 100 ns simulation. This helped in the formation of stable binding to the selected receptor protein.

### 3.6. Post‐MDS Thermal MM–GBSA​ Calculation

**FIGURE 11:**
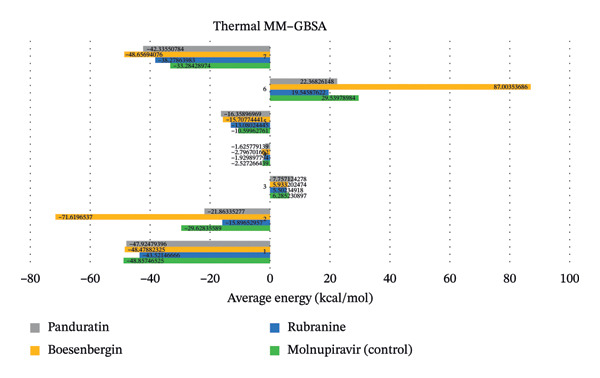
Postsimulation binding free energy (Thermal MM–GBSA) calculation of each ligand compared to molnupiravir is color‐coded using the following scheme with the targeted protein.

The thermal_mmgbsa.py Python tool was used to assess the binding free energies of the receptor–ligand complexes during the 100 ns MD. MM–GBSA analysis was performed on each of the 20 distinct snapshots of the Desmond MD trajectory. A more negative binding free energy (MM–GBSA_dG_Bind) indicates a stronger interaction between the ligand and the receptor. Among the selected compounds, molnupiravir (ΔG_bind = −48.857 kcal/mol) and boesenbergin (ΔG_bind = −48.47 kcal/mol) demonstrated stronger binding energies compared to rubranine (ΔG_bind = −43.52 kcal/mol) and panduratin (ΔG_bind = −47.924 kcal/mol) (Figure 11).

## 4. Discussion

In silico drug design has emerged as one of the most significant and efficient methods in early‐stage drug discovery by reducing cost and time with greater precision. Therefore, to accelerate the development of effective medicines for the SARS‐CoV‐2 virus, we evaluated the outcomes of multiple processes in a computer‐simulated environment [40, 47–49]. PLpro of SARS‐CoV‐2 is one of the most intriguing therapeutic targets for the virus because it possesses deubiquitination and deISGylation activities and has major implications on innate immune response during infection [50, 51].

The AutoDock Vina tools were used in blind docking to evaluate each drug candidate and identify those with the most stable and meaningful scores. Rubranine, boesenbergin B, and panduratin A were discovered to have lower binding affinities and greater binding energies with the PLpro protein. Our identification of the potential noncatalytic pockets is supported not only by docking but also by postdocking analysis, prime MM–GBSA, extensive MD simulations, and post‐MD thermal MM–GBSA calculations yielding consistently favorable binding free energies.

Previous reports have identified panduratin A, boesenbergin B, and rubranine as compounds that may be useful in preventing the action of several viruses. Panduratin A has been experimentally shown to be effective against SARS‐CoV‐2 infection by interfering with both viral entry and post‐entry replication stages [52]. Although the antiviral data on boesenbergin B and rubranine are limited, the flavonoid‐type natural compounds have been widely reported as having potential antiviral properties, including against coronavirus [53, 54].

Simultaneously, these three compounds demonstrated favorable pharmacokinetic profiles, including high gastrointestinal absorption, low toxicity, and compliance with Lipinski’s Rule of Five and other drug‐likeness filters. In the subsequent phase, strong hydrophobic and hydrogen interactions between the target protein and selected ligands were observed during the binding interaction analysis. Both static and thermal MM–GBSA analyses consistently supported stable binding of rubranine, boesenbergin B, and panduratin A to PLpro, with binding free energies comparable to molnupiravir (see Table 3). Among the compounds tested, boesenbergin B showed the most favorable ΔG_bind value, followed by rubranine and panduratin A. The notably lower ΔG_bind values for the top compounds suggest stronger and more stable binding to the PLpro active site compared to the reference drug [50].

Meanwhile, the stability and compactness between the PLpro protein and the phytochemical ligand complex are confirmed through MD simulation [43, 55]. RMSD and RMSF analyses indicated that all three compounds maintain stable binding to PLpro protein, with fluctuations comparable to the reference compound [53]. Compared to control molnupiravir, compounds boesenbergin B and panduratin A demonstrated similar RMSF and RMSD results against the target protein PLpro (Figures 4 and 5) with effective potentiality for use as COVID‐19 drugs. Though the compound rubranine exhibits minimal fluctuation in terms of RMSD value, it remains within the acceptable threshold of 3 Å. By calculating the center of mass for Rg from the protein N and C terminals, the compound boesenbergin B has shown little variation, while the rest of them have higher Rg values, indicating strong compactness. The interaction of the complete protein surface with its water molecules is assessed using the total energy by region of the ligand and protein, and the value of SASA has been investigated to better understand the interaction between protein complexes [54, 56]. Rubranine has the best SASA values for suppressing SARS‐CoV‐2’s PLpro protein compared with the control drug molnupiravir. In contrast, boesenbergin B and panduratin A had almost similar SASA values to the control medication with little fluctuations (Figure 6). Additionally, all ligand compounds had greater potential values than the control medication in the MolSA and PSA validation graphs (Figures 7 and 8). Furthermore, the intermolecular interactions (ionic bonds, hydrogen bonds, water bridge links, and hydrophobic bonds) have been examined throughout a 100‐ns simulation that supports the structural stability of these PLpro–ligand complexes [48, 57].

## 5. Conclusion

Due to having meager effective medications and the recent pandemic catastrophe, there has been an urge for the development of highly sensitive and highly specific target‐based antiviral drug candidates for tackling any further outbreak by the SARS‐CoV‐2 coronavirus or its possible closer mutant strains. This study tried to find phytochemical‐based efficient antiviral drug candidates from indigenous plant metabolites against the PL^pro^ target protein through the in silico method. In this structural interaction‐based computational experiment, we have revealed three potential antiviral phytochemicals: rubranine, boesenbergin B, and panduratin A, respectively, which have raised the chances of getting closer to the discovery of the most sensitive and effective antiviral drug against SARS‐CoV‐2 throughout the animal and human clinical trials.

## Author Contributions

Conceptualization: Veeranoot Nissapatorn, Dhrubo Ahmed Khan, Mohammad Ashik Sheikh, Raihan Rahman Imon, Shamin Ahmed; data curation: Veeranoot Nissapatorn, Dhrubo Ahmed Khan, Mohammad Ashik Sheikh, Raihan Rahman Imon, Shamin Ahmed; formal analysis: Veeranoot Nissapatorn, Dhrubo Ahmed Khan, Mohammad Ashik Sheikh, Raihan Rahman Imon, Shamin Ahmed, Harasit Gharami, Ryan V. Labana, Tajudeen O. Jimoh, Anand Gaurav, Maria L. Pereira, Muhammad Nawaz, Julieta Z. Dungca, Manik Ghosh; funding acquisition: Veeranoot Nissapatorn; methodology: Dhrubo Ahmed Khan, Mohammad Ashik Sheikh, Raihan Rahman Imon, Md. Imtiaz, Shamin Ahmed, Harasit Gharami; project administration: Veeranoot Nissapatorn, Maria L. Pereira; software: Dhrubo Ahmed Khan, Mohammad Ashik Sheikh, Raihan Rahman Imon, Md. Imtiaz, Shamin Ahmed; supervision: Veeranoot Nissapatorn, Maria L. Pereira, Md. Nazmul Hasan; validation: Ryan V. Labana, Tajudeen O. Jimoh, Anand Gaurav, Maria L. Pereira, Muhammad Nawaz, Julieta Z. Dungca, Manik Ghosh; visualization: Dhrubo Ahmed Khan, Mohammad Ashik Sheikh, Raihan Rahman Imon, Harasit Gharami, Shamin Ahmed; writing–original draft: Veeranoot Nissapatorn, Dhrubo Ahmed Khan, Mohammad Ashik Sheikh, Raihan Rahman Imon, Md. Imtiaz, Shamin Ahmed, Harasit Gharami; writing–review and editing: Ryan V. Labana, Tajudeen O. Jimoh, Md. Nazmul Hasan, Anand Gaurav, Maria L. Pereira, Muhammad Nawaz, Julieta Z. Dungca, Manik Ghosh.

## Funding

This study was supported by the Plant Genetic Conservation Project Under the Royal Initiative of Her Royal Highness Princess Maha Chakri Sirindhorn—Walailak University Botanical Garden, Nakhon Si Thammarat, RSPG‐WU‐14/2567 and Project CICECO—Aveiro Institute of Materials, UID/50011/2025 (DOI 10.54499/UID/50011/2025) & LA/P/0006/2020 (DOI 10.54499/LA/P/0006/2020), financed by national funds through the FCT/MCTES (PIDDAC).

## Conflicts of Interest

The authors declare no conflicts of interest.

## Data Availability

The data that support the findings of this study are available from the corresponding author upon reasonable request.
